# Towards Bridging the Gap Between Computational Intelligence and Neuroscience in Brain-Computer Interfaces With a Common Description of Systems and Data

**DOI:** 10.3389/fninf.2021.699840

**Published:** 2021-08-23

**Authors:** Avinash Kumar Singh, Guillermo Sahonero-Alvarez, Mufti Mahmud, Luigi Bianchi

**Affiliations:** ^1^School of Computer Science, University of Technology Sydney, Sydney, NSW, Australia; ^2^Department of Mechatronics Engineering, Universidad Católica Boliviana “San Pablo”, La Paz, Bolivia; ^3^Department of Computer Science, Nottingham Trent University, Nottingham, United Kingdom; ^4^Medical Technologies Innovation Facility, Nottingham Trent University, Nottingham, United Kingdom; ^5^Computing and Informatics Research Centre, Nottingham Trent University, Nottingham, United Kingdom; ^6^Department of Civil Engineering and Computer Science Engineering, Tor Vergata University, Rome, Italy

**Keywords:** brain-computer interface, standard, functional model, computational intelligence, neuroscience, FAIR, unified-terminologies

## 1. Introduction

We are entering the era of Open Science, which is the practice of science toward encouraging collaboration, contribution over research data, research processes, tools, scripts/codes, and any other relevant information. This mere definition involves the development of frameworks that support transparency and accessibility for the knowledge generation (Vicente-Saez and Martinez-Fuentes, 2018). However, although the practice of sharing by itself comes with great benefits (Woelfle et al., 2011), particularly for the scientific community, it poses significant challenges in terms of the development of common standards among researchers.

The generation of new knowledge is inherent to novel research topics and attractive subjects and questions. Brain-Computer Interfaces (BCI) (Vallabhaneni et al., 2005) is one such field that has attracted a lot of attention among researchers. BCIs allow people to interact with the environment by directly using their brain signals, thus bypassing nerves and muscles' natural pathways. In the last two decades, several systems have been proposed and simple explorations in academic search engines, like PubMed and Google Scholar, of the term “Brain-Computer Interfaces” provide more than 3K to 40K results, respectively, with many more being published every year. This still increasing exponential research over BCIs represent a highly multidisciplinary field, in which neuroscientists, mathematicians, physicians, computer scientists, and engineers, to name a few, interact with each other to improve BCIs by proposing new neurophysiological paradigms, advanced brain signals recording methods and devices, better mathematical procedures, and state-of-the-art decoding algorithms.

There are several open data resources available to and from the BCI community. Data resources as MOABB (Jayaram and Barachant, 2018), and software tools like EEGLAB^1^ or MNE^2^ are meant to use data formats and methods such as the European data format (EDF), JavaScript Object Notation (JSON) or comma-separated values (CSV) files to make it possible to easily mix data-processing techniques even if the data were obtained from different sources. However, these and other resources still use different terminology, data formats, processing methods, and machine learning algorithms. While diversity is expected and the data workflow entirely depends on designers, some aspects—like terminology and content structure—should be uniformed to ensure reproducibility and reach wider audiences while providing a rich environment for BCI development.

Sharing data and tools does not guarantee to make them useful. The reasons underlying are related to the variety of employed BCI paradigms (Abiri et al., 2019), tools used (e.g., BCILab Kothe and Makeig, 2013, BCI2000 Schalk et al., 2004, etc.), differences between experiment environments (MATLAB, Unity, Python, etc.), and different performance (Mowla et al., 2018). Such a varying level of information coming from various researchers has created many hurdles and significant gaps in sharing, understanding, comparing, and importantly expanding knowledge in the BCI communities. For example, when researchers work on steady-state visually evoked potential (SSVEP) -based BCIs (Lin et al., 2018), it would be ideal for assuring reproducibility to provide, besides the original acquired data, information like the number of unique flickering stimuli that are presented to the user, the flickering rate, and time distribution (i.e., uniform or not), among others. Additionally, descriptions should add details from the hardware and signal processing perspectives such as the impedance of electrodes, type of reference used, applied signal filters, enable/disable DC-offset flag, and even appropriate labeled data produced by peaks detection algorithms, etc. These details may let researchers assess conclusions properly in an unbiased manner and accelerate the advancement of BCI technologies.

## 2. Challenges and Open Issues

Imagine for a brief moment that a neuroscientist researcher would like to analyze BCI data and begins to examine the literature and similar previous studies. Unfortunately, as data formats are different, more time is spent on trying to understand how to extract and visualize the data than in understanding the principles of the underlying BCI experiments or concepts. On the other hand, a machine learning engineer would also like to test a new method for BCI, but the computed results are non-consistent. Partly, this may be due to misunderstanding the physiological principles that suppose each of the experiments, e.g., some of them might be using event-related potentials (ERP) while others Mental Tasks.

The fact of having multidisciplinary approaches into the BCI design process is enriching, however, it also adds challenges that emerge because of background differences. While computer scientists and computational intelligence researchers may find it easy to handle data, researchers with a neuroscience background may struggle when doing it. Similarly, neuroscience researchers might understand concepts related to the physiological foundations of BCI more fluently, but machine learning engineers may need to learn these concepts from scratch.

The vast amount of datasets that can be used for BCI research do not follow a standard structure of information, thus, some datasets may include more information than others. For example, some of them include references to employed psychological questionnaires, but not explaining too many technical details like in Cho et al. (2017), others—like the datasets included in the BNCI website^3^—follow a more descriptive structure. This lack of common format makes it difficult to understand what neurophysiological concepts were used and visualize the data to further explore its structure.

The gap that arises from this context is unavoidable. Nevertheless, it is possible to propose tools that can contribute to close it by first identifying the challenges. Questions as: what file format to use, what information should be stored, how do we make data more accessible to everyone, and how can we guarantee reproducibility must be effectively addressed to ensure the continuous development of BCI research within the framework of Open Science.

To answer these and other questions, the IEEE Standard Association P2731 Working Group was established in 2019, following a Conference Workshop discussion (Bianchi, 2018) to develop a standard for a unified terminology, data storage, and functional model for BCIs to allow an effortless and effective sharing of data and tools among neuroscientists, data scientists, users or BCI enthusiasts^4^. The authors of this manuscript are active members of it and invite interested readers of this manuscript to join them.

## 3. Existing Frameworks to Describe BCIs

**Figure 1:**
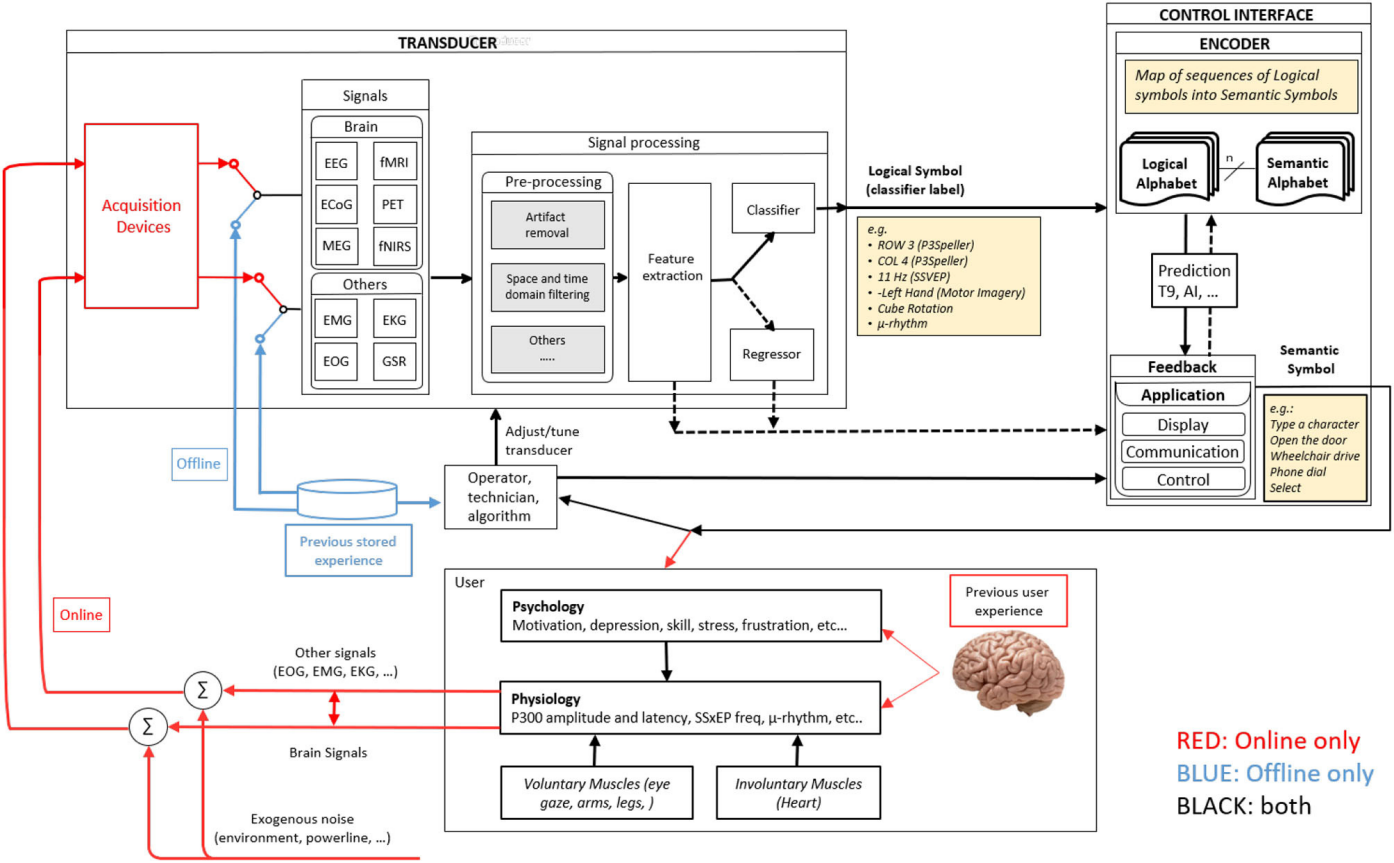
The work-in-progress BCI functional model of the IEEE P2731 Working group. Extracted from Easttom et al. (2021) under License CC 4.0.

Practices over BCI data management are partly related to developed frameworks. Previous attempts to build a common framework for describing BCI structure and working principles exist through significant works or deliberate proposals. For example, Vidal's approach to employing brain signals produced one of the earliest structural BCI's definitions: experiment protocol, signal acquisition, control, and processing (Vidal, 1973). Further, Mason and Birch proposed a general framework by defining a functional model that covers stages as experiment execution, feature extraction and translation, control, and device interface (Mason and Birch, 2003). The layout stated by both works has not changed significantly over the years. In fact, recent contributions—as those proposed in Wolpaw and Wolpaw (2012) and Nam et al. (2018)—state similar constitutions—as the one proposed by Easttom et al. (2021) shown in Figure 1, with the only difference of including more detail in the definitions due to the continuous field evolution.

A common standard definition of BCI elements follows the need to express how systems are built and used. From this perspective, it's not enough to define what functional components a BCI includes, but instead focus also on what information should be provided to the researcher and how it must be structured. Details as to the type of employed biosignals, acquisition devices, the number of channels or sources, sampling rates, among other technical related information, are required to provide more insight to the researcher regarding technical considerations. However, aspects related to the neurophysiological phenomena on which the BCI is based, the used protocol for the experiment, or even the psychological features of the subjects should be considered as well. Moreover, and as stated before, the diversity of backgrounds of each BCI researcher makes the data publishing stage difficult, as formats and data arrangement patterns may differ from one to another.

In the past, few works have focused on how a BCI should be described from the format or data arrangement pattern perspective. One of them is proposed by Quitadamo et al. (2008) and aims at using UML to describe more accurately a BCI. Similarly, Gorgolewski et al. (2016) proposed the Brain Imaging Data Structure (BIDS), a standard to capture the metadata information required for commonly used software in MRI data, and which later is complemented in Pernet et al. (2019) to establish the same principles over EEG data. Finally, XML-based Clinical Experiment Data Exchange schema (XCEDE) is another approach that uses eXtensible Markup Language (XML) to provide a hierarchical description of a dataset and that could be used to structure BCI related information. The reader must note, however, that from all listed formats, not all of them are thought to be used exclusively in BCI and, therefore, the complete applicability to the particular scenario that this technology implies is not assured.

## 4. Proposed Framework

Although multiple data formats have been proposed, they still suffer from issues that can not overcome the gap between computation intelligence researchers and neuroscience. There is a need for another kind of structure specifically designed for the communities mentioned before. In this article, we want to stimulate a discussion among the community to work on better and unified standards that can benefit everyone as per FAIR (findability, accessibility, interoperability, and reusability) principle. According to FAIR principal, BCI data should be recorded and stored in a way that emphasizes computational intelligence researchers to easy to find, access, interoperate and reuse data with minimal intervention and any domain-specific knowledge. Therefore, encourage to overcome the gap between computational intelligence researchers and neuroscientists. Majorly three important aspects should govern the process of developing a suitable data formats:

1. Address the needs of a computational intelligence community working in BCI,
2. Address the needs of a neuroscientist, and
3. Be interoperable according to the FAIR principles (Jansen et al., 2017).

Several hurdles need to be overcome to develop such a data format, such as varying terminology across different researchers. The varying terminology does not only create confusion among neuroscientists, but is troublesome to non-domain experts such as computational intelligence researchers. For example, P3, P300, positivity; all of them represent closely similar phenomena, which is a positive peak at around 300 ms in ERP (Abiri et al., 2019). Another major difficulty is from the computational intelligence community, which has different standard metrics to evaluate algorithms that are not comparable to each other in several cases. Similarly, the intersection of both computational intelligence and neuroscience researchers requires clear and accessible definitions of concepts as information transfer rate, signal-to-noise ratio, computation cost, etc.

Current efforts to develop standards are justified through the desired reproducibility of BCI studies and increase resource accessibility for researchers who do not work exclusively on the topic. Making such a standard lets resources to be easily shareable and provides the same platform following the FAIR principle. Therefore, adherence to community standards, attention to crucial metadata and workflows, and the promotion to follow standard practice ensure credit to investigators and truly help new knowledge grow in a robust, data, and resource-driven ecosystem.

## 5. Ongoing Efforts

There are several initiatives currently running to overcome the FAIR problem between computational intelligence and neuroscience society. Some of them are as follows:

### 5.1. Neurodata Without Border

It is an initiative to provide a common standard to neuroscientist to share, archive, use, and build analysis tools for neurophysiology data by adopting a unified data format^5^, although not entirely focused on BCI.

### 5.2. IEEE P2731 WG Initiatives

The activity and progress of the P2731 WG have been illustrated and discussed at several events in the last two years, such as the BCI Online Thursdays of the BCI Society, as well as the IEEE WCCI 2020, the IEEE SMC 2019, and the IEEE EMC 2019 Conferences to name few. An online survey is also available at the following link^6^ dealing with data storage to stimulate the discussion and then moving toward the definition of a standard file format for BCIs^7^.

### 5.3. The Neuroimaging Data Model

This initiative is taken by NIH Brain Initiative to overcome inconsistent terminologies, description of the design and intent of an experiment, experimental subject characteristics, and the data acquired. This initiative aims to improve data reusability, comparison, integration along with the adoption of the controlled vocabularies through community engagement^8^.

## 6. Conclusion

In this article, we have raised an important question to be considered following FAIR principles to minimize the gap between researchers from the community of computational intelligence and neuroscience. While it is clear that everyone may agree on the fact that a good standard could provide great advantages to the whole BCI community, it is not clear how to achieve this goal. Researchers do not want to spend time modifying their tools, methods, or data format to be standard compliant because it can be time-draining and unclear on the revenue. However, it seems also clear that the time saved by reusing data, tools, and methods shared by others is more significant. Besides, the possibility of performing analyses on larger datasets, such as those that could be created by merging data from different labs, will produce results with more statistical power. It is then of fundamental importance to achieve standards in the BCI research, a fact that can reasonably occur over time and in different steps, such as for allowing offline analyses or online interoperability among different tools. In both cases, there is the need to define file formats for the brain signals, for the paradigms, for the classifiers, for the performances. This could be achieved in a reasonable amount of time and could show to the people that adhering to the standards will provide more pros than cons. We have provided an example of a framework that could be adopted by the community to store BCI related data. Nevertheless, the first step is to realize that it is of fundamental relevance to start the discussion on BCI standards, possibly by contributing to one of the actions that are actually active.

## Author Contributions

All authors listed have made a substantial, direct and intellectual contribution to the work, and approved it for publication.

## Conflict of Interest

The authors declare that the research was conducted in the absence of any commercial or financial relationships that could be construed as a potential conflict of interest.

## Publisher's Note

All claims expressed in this article are solely those of the authors and do not necessarily represent those of their affiliated organizations, or those of the publisher, the editors and the reviewers. Any product that may be evaluated in this article, or claim that may be made by its manufacturer, is not guaranteed or endorsed by the publisher.

## Footnotes

^1^ https://eeglab.org/

^2^ https://mne.tools/stable/index.html

^3^ http://bnci-horizon-2020.eu/database/data-sets

^4^ https://standards.ieee.org/project/2731.html

^5^ https://www.nwb.org/

^6^ https://forms.gle/Gs1yF8TXVpD5d9yQ6

^7^ https://standards.ieee.org/project/2731.html

^8^ https://braininitiative.nih.gov/
